# Setting national research priorities for difficult-to-treat depression in the UK between 2021-2026

**DOI:** 10.7189/jogh.12.09004

**Published:** 2022-12-22

**Authors:** Natalya Chapman, Michael Browning, David Baghurst, Matthew Hotopf, Debbie Willis, Stuart Haylock, Sana Zakaria, Jan Speechley, James Withey, Edmund Brooks, Fiona Chan, Sofia Pappa, John Geddes, Lisa Insole, Zeid Mohammed, David Kessler, Peter B Jones, Parisa Mansoori, Difficult to Treat Depression Research Priority Setting Group, Saiqa Ahmed, Ian M Anderson, Michael Browning, John Campbell, Jonathan Cavanagh, Philip Cowen, Breda Cullen, Alistair Davies, Riccardo De Giorgi, Chris Dickens, Nicholas Graham, Neil Harrison, Keith Hawton, Lisa Insole, Peter B Jones, David Kessler, Glyn Lewis, Brynmor Lloyd-Evans, Steven Marwaha, R Hamish McAllister-Williams, Peter McGuffin, Andrew M Mcintosh, Valeria Mondelli, Richard Morriss, Amy O'Donnell, Sofia Pappa, Carmine Pariante, Monika Parkinson, Lisa Peppercorn, Victoria Pile, Alasdair Rooney, Giovanni Salvi, Daniel Smith, Andrew Steptoe, Rebecca Strawbridge, Argyris Stringaris, Edward Watkins, Nicola Wiles, Allan H Young

**Affiliations:** 1 National Institute for Health and Care Research Central Commissioning Facility, Twickenham, UK; 2 Department of Psychiatry, University of Oxford, Oxford, UK; 3 Oxford Health NHS Foundation Trust, Oxford, UK; 4 National Institute for Health and Care Research Office for Clinical Research Infrastructure, Twickenham, UK; 5 Department of Psychological Medicine, Institute of Psychiatry, Psychology and Neuroscience, Kings College London, London, UK; 6 South London and the Maudsley NHS Foundation Trust, London, UK; 7 National Institute for Health and Care Research Evaluation, Trials and Studies Coordinating Centre, Southampton, UK; 8 West London NHS Trust, London, UK; 9 Department of Psychiatry, Imperial College London, London, UK; 10 Cumbria, Northumberland, Tyne and Wear NHS Foundation Trust, Newcastle, UK; 11 Centre for Academic Mental Health, Bristol Medical School, University of Bristol, Bristol, UK; 12 Department of Psychiatry, University of Cambridge, Cambridge and NIHR ARC East of England, Cambridgeshire & Peterborough NHS Foundation Trust, UK

## Abstract

**Background:**

Difficult-to-treat depression (DTD) presents a substantial health care challenge, with around one-third of people diagnosed with a depressive episode in the UK finding that their symptoms persist following treatment. This study aimed to identify priority research questions (RQs) that could inform the development of new and improved treatments, interventions, and support for people with DTD.

**Methods:**

Using an adapted Child Health and Nutrition Research Initiative (CHNRI) method, this national prioritisation exercise engaged 60 leading researchers and health care professionals in the UK, as well as 25 wider stakeholders with relevant lived experience to produce a ranked list of priority RQs in DTD. The final list of 99 distinct RQs was independently scored by 42 individuals against a list of five criteria: answerability, effectiveness, impact on health, deliverability, and equity.

**Results:**

Highly ranked RQs covered a range of novel and existing treatments. The three highest scoring RQs included evaluation of psychological and pharmacological therapies (eg, behavioural activation, and augmentation therapies), as well as social interventions to reduce loneliness or increase support for people with DTD.

**Conclusions:**

This exercise identified and prioritised 99 RQs that could inform future research and funding decisions over the next five years. The results of this research could improve treatment and support for people affected by DTD. It also serves as an example of ways in which the CHNRI method can be adapted in a collaborative manner to provide a more active role for patients, carers, and health care professionals.

Despite the range of treatments currently available for depressive disorders, many individuals do not adequately respond to multiple treatment attempts [1]. Depression is a major cause of disability worldwide, affecting between 4%-10% of the population across their lifetime [2-4]. Evidence has shown that, in UK primary care settings, around 55% of patients with depressive episodes continue to experience depressive symptoms after taking antidepressants for at least six weeks [1]. Following multiple anti-depressant treatments, only two-thirds will experience even a partial response, which leaves 15%-30% of people with depression that fall into the category of treatment-resistant depression (where their condition does not adequately respond to two or more treatments) [5,6]. This can result in poorer outcomes, including an increased likelihood of relapse, higher illness burden, increased mortality, and an overall lower health-related quality of life compared to other patients who are able to receive effective treatment for their condition [7]. Besides increased distress for the individual trialling multiple treatments, this also contributes to greater economic costs which increase with the severity of resistance to treatment [8,9]. Difficult-to-treat depression (DTD) therefore poses a substantial health care challenge and represents an area in need of additional research.

Many terminologies and associated definitions are used to describe cases where depression does not adequately respond to provided treatment(s). Clinically, it is often referred to as “treatment-resistant depression” – depression that has failed to respond to two antidepressants given sequentially with an adequate dose and for an adequate duration [5,10,11]. However, this definition has been discussed at length, along with how evidence-based it is and whether it accurately represents this subgroup of patients with depression [12-15]. Through consulting with the public contributors, we concluded that an alternative terminology of DTD along with a broader definition (defined in the methods section) would be preferable to avoid excluding subgroups of the patient population [14].

Research into depression and mental health has historically not received funding proportionate to the high population burden [16,17]. Mental health research receives an average of £124.3 million of funding each year, whereas cancer research receives £611.7 million. This equates to £9 vs £228 per person with the condition, respectively [16]. The UK government and many health research funders are beginning to acknowledge the importance of research in this field and have subsequently announced new initiatives to facilitate and fund it [18-21]. To inform future funding and to ensure that research aligns with health needs, it is important to identify research priorities using a systematic and inclusive method.

There have been several recent research prioritisation exercises in mental health with have varied in approach, scope, and scale [22-26]. The recently published Mental Health Research Goals identified four high-level goals on which to focus the nation’s research efforts based upon scientific evidence and the needs of the UK health care system [22]. Given that mental health includes multiple different disorders, it is helpful to prioritise research investment at a more granular level. To our knowledge, no research prioritisation has been carried out on the specific topic of DTD (or variations of terminology for non-response to treatment in depression eg, treatment-resistant depression).

Given the high burden of depressive disorders, there is a need to advance research to support and treat people whose depression does not respond to the standard first-line treatments. This project used an adapted version of the Child Health and Nutrition Research Initiative (CHNRI method to identify the health research priorities in DTD and inform future research investments. The CHNRI’s crowdsourcing approach engages with a wide range of stakeholders including funders, policymakers, and researchers [27-29]. We tailored this method to broaden involvement from beyond the research community including people with lived experience of DTD and health care professionals.

## METHODS

### Summary of the Child Health and Nutrition Research Initiative (CHNRI) method

We adapted the CHNRI method to identify priority research questions (RQs) in DTD. Originally developed between 2005 and 2007, this crowdsourcing exercise allows for the visualisation of the research community’s collective wisdom on RQs identified as priorities for a given topic [30]. Once RQs have been submitted, scored against defined criteria, and ranked, this process identifies the strengths, weaknesses, and priority of each proposed RQ [30]. This information can subsequently be taken into consideration by researchers, funders, patients, and wider stakeholders when deciding upon future research in the selected area. The method is systematic, transparent, and easily replicable, which is likely why it has become the most popular method for health research prioritisation worldwide [31]. Additionally, it requires few resources and can be conducted relatively quickly (3-6 months) in comparison to other research prioritisation methods [31]. The adaptations made to the CHNRI methodology are described in the methods and results sections.

### Forming a management team

An appropriate expert management group consisting of 12 people was established for coordinating and steering the project. Traditionally, the CHNRI process is managed by a small team of individuals who should represent the views of investors in health research [30]. We considered it important to also include other health research stakeholders’ views to increase the applicability of the results; we invited four public contributors (individuals living with DTD or with experience of DTD) to bring different lived experience perspectives and two clinical academics (with expertise in mood disorders) to represent both the clinical and researcher communities in this exercise, alongside five other National Institute for Health and Care Research (NIHR) staff members.

### Context and criteria

For the CHNRI process to be transparent and replicable, the core elements of the context should be clearly defined and documented [30]. The final context was defined by the management group with wider stakeholder input according to CHNRI guidelines [28,30]. This step is detailed in section S1 of the **Online Supplementary Document**.

The scoring criteria were proposed by the management team based on CHNRI guidelines and are similar to the “standard” criteria suggested in the original methodology [30]. Following this, several health research funders and charities funding mental health research in the UK were invited to comment on the selected criteria and definitions to provide an opportunity to make changes where necessary. The defined context and criteria can be found in **Box 1**.

Box 1 **Predefined context for research priorities and criteria used for scoring:**

Context

- Population of interest: All adults (16+)
- Diseases, disability, and death burden: Difficult-to-Treat Depression (either uni- or bi- polar), *where a patient has tried more than one evidence-based treatment (e.g., medication or talking therapy) for unipolar or bipolar depression and has not been sufficiently helped by it.*
- Geographic limits: National (United Kingdom)
- Timescale: the results of the proposed research questions should be available within 5 years
- Purpose of research*: Research to develop new and improved treatments, interventions, and support for people with difficult-to-treat depression (in alignment with goal three of the Mental Health Research Goals)[22]

Scoring criteria

- Answerability: Some health research questions will be more likely to be answerable using the existing knowledge/research tools/capacity and in an ethical way through a well-designed study.
- Effectiveness: Some health research questions will be more likely to generate/improve truly effective interventions. Results of this research have high potential to improve health by: (1) shaping future health planning, implementation, and treatment guidelines; (2) improving delivery of health and care by improving acceptability, accessibility, suitability, efficiency, efficacy, and/or safety of treatments or services; and/or (3) improving societal and system preparedness for future health challenges.
- Impact on Health: Some health research questions will have a higher potential to generate positive outcomes for health (on either a population or individual level). Results of this research could improve health by: (1) reducing disease incidence and/or prevalence; (2) reducing social, environmental, and/or individual risk factors; (3) improving quality of life, addressing patient needs or delivering individual patient benefit.
- Deliverability: Some health research questions will be more likely to lead to/impact health interventions that will be deliverable, affordable, and scalable in the desired population and setting given resources available.
- Equity: Some health research questions will lead to health interventions that will be accessible to all (not just the privileged in the society/context), thus won’t increase inequity. Results of this research have high potential to: (1) lead to interventions or services that will be accessible and affordable to everyone, including members of vulnerable groups; (2) lead to policy, plans, interventions, or services that could reduce health inequality; (3) have additional positive effects through community involvement. This could be achieved by policies or interventions that target, empower and measure access/ uptake by vulnerable groups to reduce risk and disease exposures and/or improve access to services or interventions.

*Additional context element for this exercise. Not in original CHNRI guidelines.

### Collecting research questions

Ninety researchers were invited to submit up to five RQs addressing the defined context via an online survey. As with previous CHNRI exercises, researchers were mainly invited based on their research output in previous years, defined by bibliometrics. Additional measures were also taken to diversify the invitees’ expertise and experience levels. Full details of inclusion criteria can be found in section S2 of the **Online Supplementary Document**.

Of the 90 individuals who were invited to participate, 36 completed the survey (40% response rate). This generated a total of 127 RQs (an average of 3.5 per respondent). The questions were reviewed by the management team and edited where necessary to merge overlapping questions and ensure clarity. Six RQs were removed due to being out of scope, vague, or a clear duplicate. Forty-four RQs were then merged with at least one other question. Finally, a consolidated list of 99 research questions was produced to be sent out for scoring. Further details of this process can be found in section S3 of the **Online Supplementary Document**.

### Evaluating research questions

This stage was undertaken mostly by health care professionals and researchers who were invited to participate. In total, 90 individuals were invited to score RQs against the five criteria defined in **Box 1** above (answerability, effectiveness, impact on health, deliverability, equity) using the categorical variables of Yes/No/Unsure/Undecided to indicate whether a given RQs was likely to satisfy each of the criteria.

42 respondents scored some or all the RQs against the criteria; 35 of these were originally invited giving a response rate of 39%. The remainder likely encountered the survey through colleagues/social media as it was broadly advertised. 18 (50%) of the researchers that contributed to the first survey (proposing RQs), also contributed to this survey (evaluating RQs).

### Data analysis

#### Intermediate scores

The qualitative scores (Yes/No/Undecided/Uninformed) were converted to their equivalent quantitative values (1/0/0.5/Blank). Following this, intermediate scores were calculated for each question per criterion. These were the sum of the scores (1/0/0.5) divided by the total number of non-blank scores and then multiplied by 100 to give a percentage from 0-100. These percentages represent the likelihood that each RQs will satisfy the given criteria based on the collective opinions of the scorers. Blank scores indicate that the scorer was uniformed for that question/criterion, or they did not score it, so these are not included in the denominator [30].

The overall research priority score (RPS) was then calculated as a mean of how each question scored against each of the criteria, which in our case was 5, presented as a percentage. Full calculations and scores can be found in the data set available in the **Online Supplementary Table**.

#### Wider stakeholder engagement and weighting

To gather input from wider groups such as patients, carers, and charity representatives, we distributed a further online survey to assign weights to the scoring criteria selected, as per the CHNRI guide for involving wider stakeholders [29]. The survey was open to anyone with experience of depression, either personal experience or through family, friends, carer responsibilities, or their occupation. It was shared publicly via NIHR social media platforms and mental health charity newsletters. The survey explained the background, context, and criteria selected for this exercise and asked participants to rank the five criteria in descending order of priority from 5-1. Twenty-five individuals responded to the survey, mostly from patient/carer groups.

This group of stakeholders collectively assigned the highest average score to the criterion “Effectiveness” (4.08) followed by “Impact on Health” (4.00), “Equity” (2.72), “Deliverability” (2.36), and “Answerability” (1.84). This was calculated as an average of the criteria score (from 5-1) multiplied by its count. To convert these average scores per criterion into weights, they were divided by 3.00 which would be the average score if five criteria had an equal value.

Taking their scores into account, weighted research priority scores expressed as a percentage were calculated using the following equation as per the CHNRI guidelines [30]:



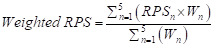



Where w_n_ is the weight for each of the criteria and the RPS_n_ is the unweighted RPS.

The weighted RPS (wRPS) was used to rank the RQs, as this reflects not only the views of the scorers, but wider stakeholders too.

#### Average expert agreement (AEA)

In addition to the above, we calculated the level of agreement between scorers according to the CHNRI guidelines [30]. This is calculated as an average of the frequency of the most common score given to a particular RQ across all five criteria, divided by the total number of scorers.



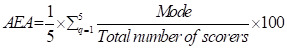



Here, q indicates the questions scorers were asked to evaluate RQs, eg, “Is this RQ likely to satisfy the following criteria?”. This is also presented as a percentage to identify RQs with the most/least agreement amongst the scorers.

## RESULTS

This exercise adapted the original methodology [30] in several ways. First, four people with lived experience of DTD joined the management group and were involved throughout the entire exercise. Second, the proposed RQs went under several iterations of review, including a collaborative workshop with patients, researchers, health care professionals, and NIHR staff members. Lastly, health care professionals and researchers were invited to score the research questions.

**Figure 1:**
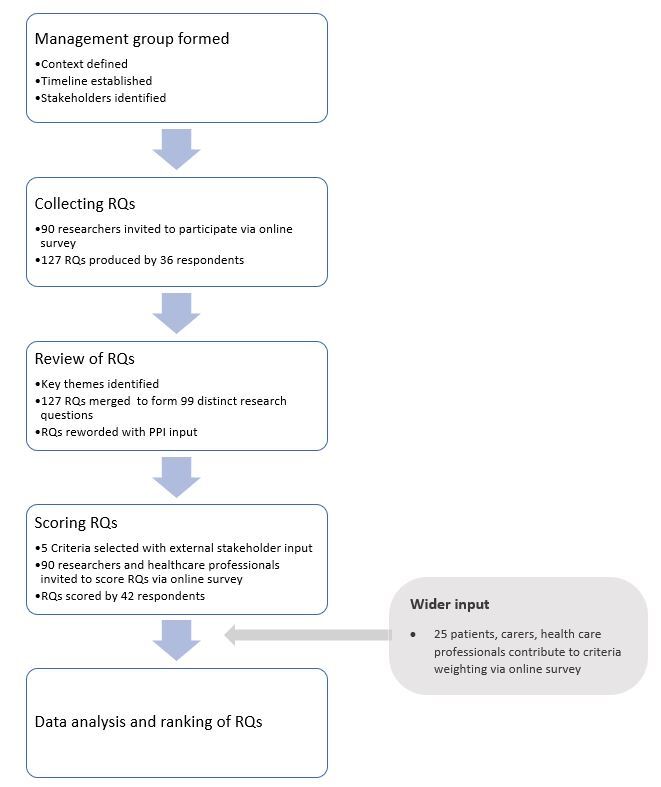
Methodology overview for setting research priorities. RQs – research questions, PPI – patient and public involvement.

In summary, 99 RQs proposed by 36 researchers were scored by 42 researchers and/or health care professionals as summarised in **Figure 1** below. Out of the 36 researchers, 18 (50%) participated in both stages of generating and scoring RQs. The full results can be found in the table of results provided in the data set available in the **Online Supplementary Document**, including the number of scorers and scores per RQ. More information on each of the survey respondents can be found in section S4 of the **Online Supplementary Document** (S4). The wRPS for each of the 99 RQs ranged from 85.5% to 47.2%, whereas the unweighted RPS ranged from 86.5% to 47.0%. The highest level of average expert agreement (AEA) was 66.7%, with the lowest being 25.2%.

**Table 1** represents the top 15 research questions ranked by the wRPS. The full set of 99 research questions ranked by wRPS, RPS, AEA, and each of the criteria, can be found in the data set available in the **Online Supplementary Document**.

**Table 1:** Top 15 research questions ranked according to wRPS

Rank	RQ	RQ #	Answerability	Effectiveness	Impact on health	Deliverability	Equity	RPS	wRPS	AEA
1	How does behavioural activation affect treatment response in DTD?	19	94	87	80	91	80	86.5	85.5	66.7
2	Is augmentation of antidepressant treatment with antipsychotics an effective treatment for DTD?	76	93	89	78	88	82	86.0	85.1	66.7
3	What programmes can be effective for increasing social inclusion and reducing loneliness for adults with DTD?	1	88	86	88	77	78	83.5	84.0	56.2
4	Does effective treatment of comorbid anxiety disorders alter the course of illness in patients with DTD unipolar depressive disorder?	48	87	87	79	80	77	82.0	81.8	58.6
5	Is lithium augmentation of antidepressant treatment more effective than placebo in people with DTD?	75	93	85	75	84	74	82.2	81.2	61.0
6	For people with DTD, what is the benefit of combining psychosocial interventions (eg, behavioural activation, social engagement, exercise) with biological or standard psychotherapeutic interventions?	65	91	83	79	81	76	81.9	81.2	61.0
7	To what extent can treatment outcomes for people with DTD be improved by provision of additional support (eg, support worker) to help people resolve life problems (eg, debt/relationship difficulties) that may be contributing to the continuation of their difficulties?	36	85	85	82	69	82	80.4	81.0	54.8
8	For people with DTD, what are the most important outcome measures to define recovery?	87	87	76	77	86	84	82.0	80.6	61.9
9	What is the risk of developing metabolic syndrome with long-term antidepressant treatment in people with DTD?	47	95	69	79	81	87	82.3	80.1	54.8
10	What are the most effective talking therapies for patients with co-morbid personality disorder/difficulty and DTD?	5	90	88	79	63	76	79.2	79.7	55.7
11	What is the effectiveness of earlier augmentation therapy in DTD vs treatment as usual?	77	90	83	79	73	74	79.7	79.5	60.5
12	What is the efficacy of psychedelic drugs for the treatment of DTD?	70	91	83	77	76	67	78.9	78.5	54.3
13	Is immunotherapy helpful for the treatment of DTD?	29	87	82	74	73	77	78.7	78.3	51.4
14	How effective is theta-burst transcranial magnetic stimulation (TMS) in people with DTD?	74	94	78	77	72	71	78.3	77.4	48.6
15	For people with DTD, should MAOIs be considered as next-step treatments where there has been a lack of response to two treatments from an initial class of pharmacological treatment (eg, SSRIs)?	16	92	77	68	78	79	79.0	77.1	51.4

DTD – difficult-to-treat depression, RQ – research question, RPS – research priority score, wRPS – weighted research priority score, AEA – average expert agreement, MAOI – monoamine oxidase inhibitors, SSRI – selective serotonin reuptake inhibitors

Overall, the wRPS is representative of the scores achieved across the criteria. The order of questions was not largely different when comparing the wRPS to the RPS or individual scoring criteria. While the order of RQs changed slightly in the weighted vs unweighted RPS, all but one of the top 15 questions are shared between the ranking (the exception being RQ74, which was replaced by RQ6 in the unweighted top 15). The highest ranking RQ according to its wRPS also fell into the top five for each of the individual criteria, apart from “Equity”, where it scores 80% placing it in the 12th position.

The top research questions included a range of different research areas and themes, including an evaluation of both existing psychological and drug therapies (eg, behavioural activation, immunotherapy, psychedelics, and augmentation therapies), as well as social interventions to reduce loneliness or increase support. Some questions in the top priorities are on patient-focused outcome measures and predictors of treatment response and outcomes, eg, RQ87: “For people with DTD, what are the most important outcome measures to define recovery?” Another subtheme which emerged in the top 15 referred to the management of comorbid/co-occurring conditions such as DTD and anxiety disorder (RQ48), or DTD and personality disorder/difficulty (RQ5).

Twelve of the top 15 priorities shown in **Table 1** relate to either existing or novel interventions. Similarly, the top three priorities all relate to evaluating interventions, including pharmacological, psychological, and social interventions. Within the top 15 identified priorities, “Answerability” was the highest scoring criterion for all questions, with the majority scoring 90% or above. The highest score across the criteria was 95% for the “Answerability” of RQ47: “What is the risk of developing metabolic syndrome with long-term antidepressant treatment in people with DTD?”, while the lowest score out of the top 15 was 63% for the “Deliverability” of RQ5 “What are the most effective talking therapies for patients with co-morbid personality/difficulty and DTD?”

The highest collective score given across all research questions was 95.5% for the “Answerability” of the 25th ranked question “Can the use of novel compounds (such as ketamine/psychedelics) in the treatment of DTD be evaluated by adequately powered Randomised Controlled Trials (RCTs)?” (RQ 60). In contrast, the lowest collective score (37.5%) given to a research question was for the ‘Deliverability’ of the following RQ: “After starting an antidepressant, what experiences in a patient's life improve the efficacy of the medication in DTD?” (RQ 68).

The highest scoring research questions per criteria were as follows:

− Answerability: “Can the use of novel compounds (such as ketamine/psychedelics) in the treatment of DTD be evaluated by adequately powered Randomised Controlled Trials (RCTs)?”
− Effectiveness: “Is augmentation of antidepressant treatment with antipsychotics an effective treatment for DTD?”
− Impact on Health: “What programmes can be effective for increasing social inclusion and reducing loneliness for adults with DTD?”
− Deliverability: “How does Behavioural Activation affect treatment response in DTD?”
− Equity: “What is the risk of developing metabolic syndrome with long-term antidepressant treatment in people with DTD?”

The AEA reflects the level of agreement amongst the scorers for the most common answer. Overall, the highest level of agreement seen throughout all the RQs was 66.7%. The top two questions ranked by wRPS, received this score. In contrast, the lowest AEA was 25.2% given to RQ4, which ranked 84th according to its wRPS. Generally, there was a negative correlation between rank (according to wRPS) and AEA, indicating that lower ranking questions had the least amount of agreement amongst the scorers, whereas generally higher scoring questions had a higher level of agreement.

The 15 lowest scoring priorities contained a broad range of questions from all five of the identified themes. Three of the lower scoring questions related to the use of genetics to stratify treatment decisions for individual patients (RQ 42, 43, and 61). All three of these scored lowest on “Deliverability” out of the five criteria and had a relatively low AEA of 36.2, 28.6, and 28.6, respectively. Similarly, a further three questions related to treatment stratification using causative factors, patient characteristics, or preferences (RQ 26, 40, and 64). Again, all three of these had an AEA lower than 37.

## DISCUSSION

This exercise aligns with national priorities outlined in the Mental Health Research Goals that were developed in collaboration with the UK’s major health research funders, charities, and people with lived experience [22]. These goals also considered the Roadmap for Mental Health Research in Europe (ROAMER) programme [25]. The James Lind Alliance (JLA) have identified more specific research questions on a range of topics within mental health over recent years, such as Bipolar, Dementia, Mental Health in Children and Young People [23]. In 2016, JLA shared the top research priorities for depression [32]. While they identified a range of priorities, only one (ranked 13th) was related to the non-response to treatment in depression: “What are the most effective ways of managing depression that does not respond to medication or talking therapies?”. Most of the priority research questions identified in our exercise would fall under this broader question identified by JLA due to overlap with our project scope. Moreover, the National Institute for Health and Care and Excellence (NICE) produced recommendations for further research within their depression guidelines published in 2009, although these are not specific to DTD [10]. The recent Lancet-World Psychiatric Association “Time for united action on depression” also identified broad areas of priority in depression for researchers, funders, and decision-makers [26].

### Key findings

This exercise aimed to assess research questions on “Research to develop new and improved treatments, interventions and support for people with DTD” in line with the Mental Health Research Goals [22]. Most questions were related to the evaluation or development of effective interventions for people whose depression does not respond well to the standard treatments. Subsequently, many of the high-ranking questions were focused on how best to manage DTD (for example, by combining different interventions). This could be due to the outlined purpose of the exercise or because the term “difficult-to-treat depression” highlights the idea of treatment (non) response. As with most definitions of DTD or treatment-resistant depression, there is a focus on describing this condition based on quantifying response to treatments [6,8,14,33-35].

The lower scoring priorities were, in general, broader, and more generic questions which also had a lower AEA. This suggests that there was a wider divergence of opinions on whether these questions would satisfy the given criteria. The amount of existing evidence may have also influenced the scoring of the priorities, in favour of interventions that have existing evidence compared to more novel interventions.

### High ranking questions

Research questions relating to combinations of treatments both across modalities (eg, pharmacological and social or pharmacological and psychological) and within modalities (eg, augmenting pharmacological treatments with other pharmacological treatments) were also ranked highly. Examples include augmentation of antidepressants with antipsychotics, lithium augmentation of antidepressant treatment, and combining psychosocial interventions with biological or standard psychotherapeutic interventions which appeared in the 2nd, 5th, and 6th ranking questions by wRPS, respectively. There is existing evidence for the use of these treatments as augmentation therapies to antidepressants and they are also recommended in NICE guidelines for depression [34,36]. Several questions based on novel pharmacological approaches such as ketamine/esketamine were also proposed, these have been shown to positively impact the treatment of depression, both in the UK settings and elsewhere [37-39]. The proposed RQs looked to examine the efficacy and side effects of these novel treatments and how they compare to alternative treatments such as lithium (RQs 12, 16, 22, 25, 55, and 60).

Besides RQs on pharmacological therapies for the treatment of DTD, many questions relating to social/psychological interventions scored highly on the impact on health, suggesting that these may be perceived as having a greater impact on the patient population. The question ranked highest for “Impact on Health” was RQ1: “What programmes can be effective for increasing social inclusion and reducing loneliness for adults with DTD?” Similarly, third in ranking for “Impact on Health” was: “To what extent can treatment outcomes for people with DTD be improved by provision of additional support (e.g., support worker) to help people resolve life problems (e.g., debt/relationship difficulties) that may be contributing to the continuation of their difficulties?”.

Several high scoring priorities were based on investigating the relationship between immunology/inflammation and DTD. A few examples include: RQ77: “Is immunotherapy helpful for the treatment of DTD?” RQ30: “Is inflammation a causal risk factor for DTD, if so, could it be a new treatment target?” and RQ92: “In patients with DTD, does a raised blood CRP (C-reactive protein) level predict response to catecholamine antidepressants?” This area received growing interest over the past few years, with emerging evidence indicating raised inflammation levels in more treatment-resistant patients with depression [40-42].

Neurostimulation therapies such as electroconvulsive therapy (ECT) and repetitive transcranial magnetic stimulation (rTMS) appeared several times throughout the list of RQs. The 2015 NICE guidelines acknowledged the use of rTMS in the management of depression due to early evidence showing no major safety concerns [43], there have also been other trials on TMS [44]. Moreover, there is evidence that ECT is efficacious in the treatment of DTD [34,45], but the question proposed for this topic (RQ69) is framed around understanding which patients it would be most effective for which falls into the theme of personalising (or stratifying) treatments.

### Lower ranking questions

The several questions related to developing or evaluating digital support/interventions did not score particularly highly. This is perhaps surprising given the increase in available digital interventions and support for people with mental health conditions, alongside the impacts of the pandemic and a move towards digital health more generally [46-48]. The highest ranked question relating to digital interventions ranked 35th: “Can self-monitoring of wellbeing for people with DTD be facilitated with digital health interventions in order to detect early warning signs of relapse?” This received its lowest score in the criterion of ‘Equity’ (RQ 56). This could perhaps reflect concerns around patients’ lack of access to technology, increasing inequity for those who are not as digitally literate, and digital interventions replacing face-to-face services [49,50]. However, other arguments in favour of digital health suggest that the use of digital technology and remote care could facilitate care for those who face challenges in attending in-person appointments [46].

Questions related to personalisation or stratification of treatments – for example using genetics – were also not scored as favourably by the scorers as other themes. Among these questions, the lowest scoring criteria was “Deliverability” (average score 58) with the highest being “Equity” (average score 70). This indicates that, while these questions might be important, they are not perceived to be deliverable, affordable, or scalable given the context defined for this exercise. Examples of these questions include RQ61 “What are the novel pharmacological treatment targets emerging from genomic (e.g., Genome-wide association study (GWAS)) and transcriptomic studies that could be used in the treatment of DTD?” and RQ43 “Does pharmacogenetics (using previously established genetic variants that predict treatment response or adverse events) help prevent difficult to treat depression or identify novel treatment strategies for people with DTD?”, both of which were ranked in the bottom five questions according to wRPS.

### Strengths and limitations

This prioritisation exercise aimed to identify the priority RQs that would be answerable, effective, deliverable, and impact health in an equitable manner over the next five years. The questions should be relevant to our outlined context and should have an overall aim to develop new and improved treatments, interventions, and support for adults with DTD. A key strength of this study is that, to our knowledge, this is the first research prioritisation exercise conducted in the UK on DTD (or TRD). There remain large challenges around identifying and managing the population of patients whose depression does not respond to the standard available treatments as highlighted by the diverse set of priority research questions proposed. This set of priority questions proposed reflects some of the common challenges and areas of opportunity.

The CHNRI method has several advantages over other commonly used health research prioritisation methods including its simplicity, flexibility, and transparency [31]. It can be conducted relatively quickly due to the use of online surveys – this exercise took around six months to complete. Online surveys also enabled engagement with many experts without a significant time burden for them and meant that their responses were completely independent, thus avoiding the risk of individuals influencing the group decision as might be the case in workshop style prioritisations.

Another strength of this exercise is the methodology adaptations implemented to give a more active role to patients, carers and health care professionals who are likely to be directly impacted by the results of the research proposed. Gaining these perspectives is important to ensure the results of any prioritisation exercise (and subsequent research) would be relevant, acceptable, and fundable [51-53]. Given this, the inclusion scope was broadened to better reflect the views of groups beyond researchers. The engagement with researchers adhered to the guidance and examples outlined in the CHNRI methodology [27]. However, we also encouraged snowballing by asking those who were contacted to invite colleagues with knowledge in the field to participate in the surveys. This was particularly relevant for the prioritisation survey where we aimed to involve health care professionals without research backgrounds. These individuals would be harder to identify using bibliometrics, for example.

Additionally, several of the health research funders and charities that fund mental health research in the UK contributed towards selecting and defining the scoring criteria with the aim that the research questions’ scores would be aligned with their funding processes. This follows recommendations in the CHNRI methodology, indicating that it is important to involve this stakeholder group throughout the exercise and that funder-supported criteria should be incorporated to ensure that the output supports their decision-making [28]. Twenty-five wider stakeholders (mostly patients/carers) were invited to assign weights to the scoring criteria, as recommended in the CHNRI guidelines for involving stakeholders [29]. Only a minority (24%) of previous CHNRI exercises had appropriately engaged with a group of stakeholders during the process [28]. While this enabled their collective views to influence the final ranking of the RQs, ideally, we would have engaged with many more stakeholders for criteria weighting to increase its accuracy. Additionally, it may be argued that different criteria may be considered more important to individual stakeholder groups, for example, “Deliverability” for research funders. In this case, the full results (provided in the data set available in the **Online Supplementary Document**) can be filtered and re-ordered according to differing stakeholder needs and interests.

The basis of the CHNRI is dependent upon the concept of the “Wisdom of Crowds” and its crowdsourcing approach; its central idea is that in most cases, a diverse and large group of independently deciding individuals will lead to better predictions than a single expert [54,55]. Previous statistical analysis has shown that the stabilisation of the scores (the point at which point additional scorers would not change the results of the exercise) occurs when there are around 45 scorers, although a sample size of 15 experts already shows reproducibility. As there were more than 40 scorers for this exercise, this would still be sufficient to give robust results, although it would be desirable to have a greater number of scorers to ensure that there are stable rankings that would not fluctuate with further scorers [56].

The findings of CHNRI exercises may be biased, as they represent the views of a limited number of involved researchers and potential plausible RQs may not have been proposed. The list of priorities will not include all potential RQs in this topic (which would be limitless) [57] and other priority research areas might not have been proposed in this exercise. To identify participants, we used a range of methods to capture both the UK’s leading researchers in this topic area and less established researchers with the aim of reflecting the views of the wider research community. Despite our best efforts, there may have been other methods to select participants, and inevitably, we may have missed certain groups of individuals that could have provided valuable contributions.

Moreover, the exercise was limited to UK-based researchers only, although many of the findings will be relevant to the international research community. We also aimed to capture a wide range of disciplinary backgrounds in an attempt to balance the sample composition; we were mostly successful, as the respondents comprised psychologists, psychiatrists, mental health nurses, and neuroscientists. Nonetheless, most of the respondents who proposed and scored the RQs were psychiatrists and psychologists (section S4 in the **Online Supplementary Document**). Psychiatrists made up most of the responses for both survey 1 (generating RQs) and 2 (scoring RQs) which represented 61% and 64% of the respondents, respectively. There may be varying schools of thought between different academics and health care professionals, with possibly different preferences for either more pharmacological or psychotherapy-based approaches. This may have impacted both the research questions proposed and the allocated scores. Nonetheless, the research questions, particularly those seen in the top 15 in **Table 1**, cover a range of different interventions suggesting that there was some balance between these differing approaches.

Despite the relatively narrow scope for this exercise, we achieved a similar response rate to that seen in other CHNRI exercises of ~ 40% [54]. Most individuals who did not participate did not respond to the invitation. For those who did respond but declined the invitation, the most common reasons were lack of time to complete the survey and/or inability to take on additional projects, or lack of expertise in the selected topic area. These mostly applied to the scoring survey, which was significantly more time-consuming than generating RQs. The greatest AEA, which reflects the level of agreement amongst the scorers for the most common answer, was 66.7%. While not too dissimilar from other applications of the CHNRI method, a higher AEA would have shown a stronger consensus around the research priorities produced in this exercise [58,59].

A review of the first 50 conducted CHNRI studies found a redundancy rate in the list of questions initially proposed of around 50% [54]. While we did not find this much duplication, the question set was consolidated from 127 to 99 by removing questions which overlapped significantly. The questions went through several reviews, rewordings, with careful consideration before being distributed for scoring. Only six questions were removed from the original list of questions, indicating a broad pool of research questions addressing the context outlined for the exercise. As indicated in section S3 in the **Online Supplementary Document**, the most saturated theme in the consolidated list of RQs was “interventions”, followed by “stratification”. Nine RQs from both themes were removed from the list and merged with other RQs. The consolidated list of questions still has some degree of overlap and inconsistency in phrasing, which is most likely unavoidable without significantly compromising the original meaning of the research question. While we hope that the final questions remained true to the full list of those proposed, it is difficult to guarantee that the original meaning of the questions was not unintentionally altered. While this exercise produced an extensive set of specific RQs, the relatively narrow context (specifically, the five-year timescale for research) may have restricted participants in generating research questions and limited their scores on criteria such as answerability and deliverability. Additionally, its focus was on later-stage translational or applied health research rather than experimental and discovery research. Lastly, another limitation may be that some of the priorities proposed may have been answered to some degree or be in the process of being funded which should be taken into consideration during further application of this study’s findings.

## CONCLUSION

We have conducted the first national priority setting exercise to identify priority research questions that could lead to the development of new and improved treatments, interventions, and support for people with DTD. The exercise engaged 60 leading researchers and health care professionals, as well as 25 wider stakeholders (mostly consisting of patients/carers) to produce a ranked list of 99 research questions scored against five criteria important to major UK funders of mental health research. Our findings suggest areas of research to develop new and improved treatments, interventions, and support for people with DTD that should be prioritised in the coming years. The results of this exercise can inform funding in this area of mental health research over the next five years in the UK.

This study also serves as an example of ways in which RQs could be systematically and transparently prioritised in a UK-based funder setting using an inclusive approach that accounts for the views of patients, carers, researchers, health care professionals, and funders, amongst others.

## Additional material


Online Supplementary Document
