# Supplementary material for: Setting national research priorities for difficult-to-treat depression in the UK between 2021-2026
Source: J Glob Health. 2022 Dec 22;12:09004. doi: 10.7189/jogh.12.09004 (PMC9727823; doi:10.7189/jogh.12.09004)
Supplement: Online Supplementary Document [file jogh-12-09004-s001.zip › jogh-12-09004-s001.pdf]

ONLINE SUPPLEMENTARY DOCUMENT

Title: Setting national research priorities for Difficult-to-Treat Depression in the UK between 2021-2026

Authors: Natalya Chapman, Michael Browning, David Baghurst, Matthew Hotopf, Debbie Willis, Stuart Haylock, Sana Zakaria, Jan Speechley, James Withey, Edmund Brooks, Fiona Chan, Sofia Pappa, John Geddes, Lisa Insole, Zeid Mohammed, David Kessler, Peter B. Jones, The DTD Research Priority Setting Group & Parisa Mansoori

S1. Defining the context and criteria

The context was defined at the start of the exercise by the management group, taking into consideration national areas of need and research priority areas.[1] The scoring criteria are used in the second stage of the CHNRI process for evaluating the research questions. In this exercise, the five scoring criteria were selected and defined with input from representatives of major funders and decision makers including: MQ Mental Health Charity, McPin Foundation, the Department of Health and Social Care, The National Institute for Health and Care Excellence (NICE), Medical Research Council, Wellcome Trust, and Johnson & Johnson. Their feedback was incorporated into the selected criteria and definitions which was finalised by the management group, according to the process outlined in the CHNRI guidelines.[2]

S2. Identifying survey participants

The approach to selecting individuals to participate in the exercise can be flexible provided that the approach is justifiable.[3]

In summary for the first survey, researchers were invited for belonging to one or more of the following groups:

- Top 50 Highest Citations in RCDC "Depression" in the past 20 years
- Top 20 Highest Publications in RCDC "Depression" in the past 5 years
- NIHR Academy Award Holder in “treatment of depression” in the past 5 years
- NIHR Mental Health Translational Research Collaboration work stream representative for Treatment Resistant Depression

As this was a national exercise, searches were limited to UK based researchers only. The invitees were invited to share invitations to other leading researchers to participate.

After removing duplicate names, 90 individuals were invited to propose 3-5 RQs via an online survey. This was followed up with several reminders and a deadline extension for those who had not yet responded. This survey was open for a total of three weeks.

For the second survey:

The researchers invited to participate in the first survey, were also invited to participate in the second stage of evaluating the proposed research questions against criteria via a separate online survey unless they had opted out. In addition to this, the management team decided it would be beneficial to invite health care professionals to score the research questions. Their engagement is important as they have a thorough understanding of the health care system, medical unmet need, patient population and are likely to be responsible for delivering the research outputs to people with DTD. The 90 individuals invited were also asked to share the survey amongst relevant colleagues as part of a snowballing technique. For this stage, NIHR and other social media sites were utilised to reach a wider audience.

Again, for this survey an invitation email was sent along with several reminders and deadline extensions for those who had not responded. This survey was open for 38 days in total. For both surveys, invitees were provided the option to opt out of participation and/or future correspondences.

S3. Reviewing research questions

The questions were first reviewed internally by members of the management group. Research themes were identified from this original list of RQs and subsequently, questions that significantly overlapped were merged. In our final set of 99 research questions, the questions were distributed across the themes as follows: Understanding DTD (11 RQs), care pathways (11 RQs), interventions (48 RQs), research methods (5 RQs) and stratification (24 RQs).

The public members of the management group were invited to reword research questions as necessary to ensure that the questions were accessible to non-research audiences. Where questions were unclear, we also asked the researchers for clarification over their questions. As a further step to ensure that the questions were clear and distinct, we held a collaborative workshop with the management group and additional health care professionals to review the process taken to consolidate the list of questions and finalise the list. The full list of 127 RQs was consolidated to 99 distinct RQs.

S4. Survey respondent roles and backgrounds

The survey respondents for generating and scoring research questions were split into three categories, Psychiatrist, Psychologist and Other. 'Other' mostly consisted of neuroscientists, non-clinical researchers, and mental health nurses. This information was estimated mostly from academic profiles or other internet resources where this was unavailable. The respondents to survey 1 and 2 also self-reported their geographical location (according to the NHSE regions as well as the devolved Nations).

For **survey 1** (generating research questions) there were 36 respondents from the following roles and geographies:

Role	Count	Proportion (%)
Psychiatrist	23	64
Psychologist	5	14
Other	8	22

Region	Count	Proportion (%)
East of England	4	11%
London	8	22%
Midlands	4	11%
North East and Yorkshire	5	14%
North West	1	3%
Northern Ireland	0	0%
Other (please specify)	1	3%
Scotland	3	8%
South East	3	8%
South West	7	19%
Wales	0	0%

For **survey 2** (scoring research questions) there were 42 respondents from the following roles and geographies:

**Role**	**Count**	**Proportion (%)**
Psychiatrist	28	67
Psychologist	6	14
Other	8	19

**Region**	**Count**	**Proportion (%)**
East of England	2	5%
London	11	26%
Midlands	6	14%
North East and Yorkshire	4	10%
North West	2	5%
Northern Ireland	0	0%
Other	1	2%
Scotland	6	14%
South East	4	10%
South West	5	12%
Wales	1	2%

For **survey 3,** which engaged with wider stakeholders, the respondent's backgrounds were self-reported. The 25 respondents reported belonging to one or more of the following groups. Five individuals selected two options for role/affiliation.

**Role/affiliation**	**Count**	**Proportion (%)**
Patients	16	53
Carers	7	23
Health care professional	3	10
Non-clinical researcher	2	7
Mental health charity representative	1	3
Clinical academic	1	3

1 Wykes T, Bell A, Carr S, Coldham T, Gilbody S, Hotopf M, et al. Shared goals for mental health research: what, why and when for the 2020s. Journal of Mental Health. 2021;1–9.

2 Rudan I, Gibson JL, Ameratunga S, Arifeen S el, Bhutta ZA, Black M, et al. Setting Priorities in Global Child Health Research Investments: Guidelines for Implementation of the CHNRI Method. Croat Med J. 2008;49:720.

3 Yoshida S, Cousens S, Wazny K, Chan KY. Setting health research priorities using the CHNRI method: II. Involving researchers. J Glob Health. 2016;6.
